# EMERALD—Exercise Monitoring Emotional Assistant

**DOI:** 10.3390/s19081953

**Published:** 2019-04-25

**Authors:** Jaime A. Rincon, Angelo Costa, Carlos Carrascosa, Paulo Novais, Vicente Julian

**Affiliations:** 1 Departamento de Sistemas Informaticos y Computación, Universitat Politècnica de València, Valencia 46022, Spain; jrincon@dsic.upv.es (J.A.R.); carrasco@dsic.upv.es (C.C.); 2 ALGORITMI Center/Department of Informatics, University of Minho, Braga 4704-553, Portugal; acosta@di.uminho.pt (A.C.); pjon@di.uminho.pt (P.N.)

**Keywords:** cognitive assistant, wearable, emotion detection, signal processing, elderly well-being

## Abstract

The increase in the elderly population in today’s society entails the need for new policies to maintain an adequate level of care without excessively increasing social spending. One of the possible options is to promote home care for the elderly. In this sense, this paper introduces a personal assistant designed to help elderly people in their activities of daily living. This system, called EMERALD, is comprised of a sensing platform and different mechanisms for emotion detection and decision-making that combined produces a cognitive assistant that engages users in Active Aging. The contribution of the paper is twofold—on the one hand, the integration of low-cost sensors that among other characteristics allows for detecting the emotional state of the user at an affordable cost; on the other hand, an automatic activity suggestion module that engages the users, mainly oriented to the elderly, in a healthy lifestyle. Moreover, by continuously correcting the system using the on-line monitoring carried out through the sensors integrated in the system, the system is personalized, and, in broad terms, emotionally intelligent. A functional prototype is being currently tested in a daycare centre in the northern area of Portugal where preliminary tests show positive results.

## 1. Introduction

Currently, society is experiencing a shift in the traditional age distribution scheme. The report [1] shows that the aging population is growing fast; in some countries, it surpasses the number of births. This leads to an aging society that will experience a decay in the number of people that is available to work and contribute monetarily to the society. Ten years ago (1999), the European Commission has presented a study that showed the economic impact of the this trend [2]. According to this study, the expected economic burden to the elderly, families and/or the state will surpass the positive influx, consuming about 20% of a country’s GDP [2]. Moreover, in terms of the family nucleus, not being economically solvent may lead to the absence of care provision and abandonment of the elderly.

Aggravating these social issues is the need of more complex caring services. Recent studies have found that the prevalence of Alzheimer’s (and other dementia diseases) is directly related to the increase of elderly people [3]. In fact, it is expected that in 2050 the number of Europeans that have some sort of dementia will rise to 18.7 Million. This means that the dependency in specialized care is growing. Apart from the aid they actively need, their medication intake (as they tend to forget) and medical control (to verify if the medication has positive or negative impact) has to be under strict supervision, as the continuous absence of medication can rapidly worsen the health condition [4]. One of the possible solutions is to promote alternative care systems, for instance, care at the elderly home. There are several benefits that come from keeping the elderly at their homes, e.g., they are in an environment that is familiar, they have to be less transported (which increases fall risks), their relatives and neighbours are able to visit them. However, there are other factors that have to be taken into account as the transition of professional care to assisted care is not direct [5]. Nonetheless, Ref. [5] refers to the fact that the benefits of keeping the elderly people at their homes as long as it is possible that it has great benefits, being one of the greatest downsides the lack of training that the assistants may have, as referred to by [6].

One of the executional issues of keeping elderly people at their home is the stagnation and repetitive and unchallenging tasks, which are often associated with cognitive problems like dementia [7]. A sign of early stages of Alzheimer is confusion, disorientation and repetitiveness; thus games and complex varied tasks are recommended to prevent and rehabilitate Alzheimer’s disease [7]. One way to overcome this issue is to introduce *Active Aging* in the elderly person’s life and daily routine. The World Health Organization has defined the concept of *Active Aging* as “...the process of optimizing opportunities for health, participation and security in order to enhance quality of life as people age” [8]. Major European institutions are focusing on this concept [9]. Thus, it is foreseeable that, in the near future, *Active Aging* will have high importance.

Although it is a rather novel concept and its usage is still far from ideal, empirical evidence reinforces the usage of *Active Aging* [10], as the acceptance by the elderly of this concept may change their life [10]. *Active Aging* promotes a health living that is founded with the pillars of continuous engagement in activities (social or otherwise) that have complex cognitive and/or physical traits. These activities help the performers exercise their body and their mind, while boosting their happiness. The overarching goal is to increase the wellbeing levels of the elderly, making their everyday life as smooth as possible. Thus, to achieve this, and taking into account the cost, the usage of technological solutions may be used, tackling all presented issues. Thus, there has to be an understanding of the elderly people’s needs and how to improve their daily life [11].

To this, some solutions have been designed, aiming to help elderly people with their activities of daily living (ADLs), in the form of digital personal assistants, explained in depth in Section 2. Nonetheless, most of these projects fail to address one important aspect, *emotion*. Older people tend to attend to their emotional status and frequently report positive emotions, the number being significantly higher than the ones reported by younger people [12]. Thus, their psychological state (thus the emotional state) may influence and create a difference between those who are active (showing happiness, less distress, optimism, loneliness or neuroticism) and those who are less active. This is even more evident as the elderly have more often health-related problems than not. Thus, the usage of emotions to influence the operation of the technological solutions is imperative.

The **EMERALD** project aims to introduce the emotions prism in the decision-making process, considering the emotional response to the suggestions of the system and correcting it when there is a negative emotional response. EMERALD is a system comprised by hardware (a sensing platform) and software (emotion detection and decision-making) that combined produces a cognitive assistant that engages the users in *Active Aging* through the suggestion of activities allowing them to follow a healthy lifestyle. It uses artificial intelligence to produce an emotional representation and, using user profiles, adjusts the suggestion mechanism to produce activity suggestion that boosts happiness levels. By continuously correcting the system according to each user emotions, the system is personalized, and, in broad terms, emotionally intelligent.

This paper is structured in the following way: Section 2 presents the related work (in the form of software or robotic assistants); Section 3 presents the system description (detailing the hardware and software components); finally, Section 4 presents the conclusions and the future work proposals.

## 2. Related Work

Assistants, in technological terms, are not a new concept. They appear in several different areas, with more or less success or appeal. As the name indicates, typically, assistants are designed to guide humans through a process or processes that they are unknowledgeable about, or to aid them in performing some tasks. The most relevant (and related) assistants are presented below in an effort to display the state-of-the-art developments in this area.

### 2.1. Robot Assistants

In the vast area that is robotics, there is currently active development of social robotics, with the aim of being used by humans in a social environment. The most known developments (due to their physical visibility and the society awareness) are in the form of robot assistants, as considered by Martinez-Martin [13].

Blue Frog Robotics (Paris, France) have developed the Buddy robot [14], which is an emotional robot, meaning that is able to demonstrate human-like emotions and perceive the emotions of the people that interacts with it. Its aim is to be a companion robot, having as its main focus human interaction. It is unable to perform tasks (apart from moving using its wheels) but has as features the ability of giving information about specific tasks and maintain a conversation fluently. The company target public for this development are the elderly and the children.

On the contrary, we have the InTouch Health [15], which is a mobile medical platform (i.e., RP-7 robot) remotely operated by a doctor. In this case, the robot is an assistant of the doctor as it is a gateway to communicate with the patient. The robot has multimedia abilities (with a screen, speakers and microphone) and some sensors (vital signs). The data gathered is sent to the tele-doctor for further analysis. This data can be used to preventively detect health problems.

A mixture of the services of the two previous robots can be found in the Sanbot Nano [16]. This robot focus on the active companionship and provides medical services to the users. The robot has the ability to perform basic diagnostics resorting to a questionnaire performed to the users, and, if needed, can call a healthcare center or directly a doctor and serve as the visual interface to them. Apart from these features, the Sanbot has a reminder ability that warns the users about medicine taking and medical appointments. When placed in a hospital environment, the robot serves as a multimedia centre that is able to maintain a light conversation with the patients, helping to establish a calm environment.

At the other end of the spectrum is the Pepper robot [17]. This robot is built to be a fully fledged assistant, being able to interact with the users in a social way. This robot has a large set of sensors and pre-programmed abilities that make it very joyful to interact. The main aim of this robot is to be used as a companion in home and commerce environments. Due to its friendly aspect, this robot has already shown to be largely accepted by the society. Being connected to the internet, it is able to maintain a fairly complex conversation and answer various queries from the users, as well as serving as a multimedia gateway using its screen, speakers and microphone. However, it should be noted that one of the main advantages that Pepper has is its development platform. Developers can enhance the robot’s features and use it for several domain-specific applications. This is the case for the PHAROS project [18] that uses the Pepper to show and evaluate medically-suggested physical exercises for elderly people.

One of the most relevant issues that robots have is their physical limitations—in this case, in terms of the electronic hardware and physical hardware. The presented robots are heavy and can make a wide range of movements, meaning that they require a large battery to operate properly and within a reasonable time, which in turn makes them heavy and cumbersome. This is an Achilles heel of the robots. Furthermore, most assistant robots are unable to carry or move objects (apart from the Pepper, which is also severely limited in terms of the weight it is able to carry), thus they are unusable for performing home tasks apart from providing instructions.

### 2.2. Cognitive Assistants

Distancing from the robotics area are the less known assistants, the cognitive assistants [19]. These assistants are mostly software-based but have the ability of using both home automation or appliances, as well as robots to interact with the users or via mobile devices. Most cognitive assistants are unassuming in terms of the usage domain or the people that use them. They are very heterogeneous in terms of the applicability.

One representative of a cognitive assistant implemented in a mobile device is the Mymemory [20]. This project focuses on aid people with Traumatic Brain Injury to remember lost memories by periodically reminding the users of activities, events or general information. The goal is to jog the memory of the users, helping them retain more information and create new brain synapses that compensate the loss of old ones. This technique has been proven successful in people with Alzheimer’s disease and with Traumatic Brain Injury in deterring the advance of the health problems. Periodically, the users are challenged by notifications presented in their smartphone to remember what they have done in previous dates, giving them hints (location, participants, etc.).

Other assistant is the PersonALL project [21], which monitors the behaviour of the elderly and displays health-related suggestions through intuitive interfaces. The aim of this project is to verify the actions of the elderly and detect if there is a decay of the motor abilities as well as critical situations like falls. As an additional feature, the system periodically interacts with the users in an effort to socialize and accompany them; this way it creates a feeling of companionship and keeps the users active and aware. An example where there is the need of a cognitive assistant to extend the abilities of people with great impairments is the module developed for the RUDO platform described in [22]. This module aims to overcome the limitations that blind people have when navigating in their home and how they interact in social situations. It works by producing notification sounds to extend the spatial and social context knowledge.

The RUDO platform is able to detect the people in the environment surrounding the blind person and where it is facing. With this information, the module produces distinct sounds to assert the social situation at each moment, thus the blind person is able to respond in a socially accepted manner.

Within the cognitive assistants, we can find different approaches that try to offer a virtual agent to facilitate interaction with older people. The project IN LIFE [23] developed a virtual Ambient Intelligence caregiver monitoring older people 24/7 in a form of a smartwatch. The virtual agent provides a range of services such as fall detection and activity monitoring. The experiments showed an improvement of the user and caregiver reactions. Another example is the work presented in [24] where results show the benefits of using virtual agents employed to assist people with cognitive limitations in managing their daily schedule and calendar. Another more recent project is that proposed in [25], where the goal is to provide person-centred care for the elderly at home, making use of current technologies. The work makes use of image processing and face recognition technologies from the generation of a 3D model of a face. Moreover, one of the main innovative aspects of this approach is its adaptive capacity to different situations and backgrounds, offering more personalized services. All of this makes older people more receptive to its use.

Taking into account the advantages of using virtual agents or avatars to improve the interaction of cognitive assistants with the elderly, other work has focused on studying key features for the design and evaluation of these virtual agents or avatars. This is the case of the work presented in [26]. In this work, authors propose to create helpful and friendly interfaces defining a correct evaluation matrix which includes visual, ambient features and performance as well as entertainment and trust elements to establish a correct relationship between the virtual agent and the elderly. The matrix helps to determine the quality issues of the developed virtual agent.

In terms of wearable emotion detection systems, The work presented in [27] proposes a platform that (using commercial hardware) is able to attain the emotional levels of the wearer, while Krause et al. presents a similar work with the main difference of using machine learning techniques to model emotion status online [28].

Nonetheless, there is still a long path until most people have a personalized assistant that attends to their disabilities and problems. We aim to close the gap by producing a cognitive assistant that is composed by both hardware and software and is able to act upon one of the most elusive human feature: emotions.

## 3. System Description

This section presents the whole system called EMERALD (Exercise Monitoring Emotional Assistant) where hardware and software have been devised to be used for generating and adapting a personalized exercises sequence for a rehabilitation process of an individual. The hardware part of the EMERALD system includes a set of bio-sensors allowing for capturing the physical stress of the person doing the exercises sequence, so that they can be used to dynamically adapt it if the system detects that it is too stressful for him. However, not only in this situation are the set of sensors also used to perceive the evolution of the emotion of the person. This emotion can also be used to adapt the exercises sequence, so the final experience obtained by each user of our EMERALD system is the most adequate exercises sequence according not only to the physical evolution of the person during the exercises sequence, but also the evolution of his emotions.

**Figure 1:**
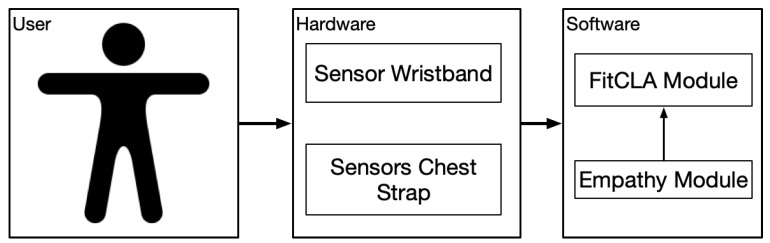
EMERALD general overview of the system’s components and their hierarchy and the information flux.

Figure 1 shows the different components forming the EMERALD system. This components can be classified in the following groups:

- User: This is the main part of the system as it is not only the source of the input sensor data, but also the goal of the actions carried out by the system.
- Hardware: This group is formed by all the different sensors that can be used to perceive the evolution of the person to the exercise sequence. In fact, this sensors are grouped into two different artefacts: the *Sensors Chest Strap*, formed by a set of sensors that will go in the user chest, and they even could be linked to a slim-fit exercise T-shirt; and the *Sensors Wristband* formed by a set of sensors collocated in a wristband worn by the user.
- Software: This group is formed by all the software modules in charge of calculating information and using this information to create and/or adapt the exercise sequence of a user according to his user profile and dynamic evolution. These modules are: *Empathy Module* according to the sensor information, calculates the current user’s emotion; and the *FitCLA* which calculates the proper exercises sequence or adapts the current one according not only to the user profile, but also his current physical and emotional stress.

In the following subsections, the hardware and software parts of the EMERALD system are detailed.

### 3.1. Hardware Description

This section presents the description of the health hardware. This assistant consists of a series of electronic devices, which allow us to acquire the signals of ECG (electrocardiography), EDA (electrical activity of the skin), PPG (photoplethysmography) and the IMU (inertial measurement unit) consisting of an accelerometer and a three-axis gyroscope.

It is necessary to take into account that nowadays there is no portable system capable of acquiring these signals in the market, which is also used for the detection of emotions. Nevertheless, it is possible to find portable systems that allow for acquiring these signals; as examples, we find the Holter [29,30] or detectors of three using GSR (Galvanic Skin Response). However, some of these devices are expensive, as is the case of Empatica [31,32,33]. In order to integrate EMERALD software into other available hardware, it is necessary to have BLE (Bluetooth Low Energy) communication and GATT (Generic Attribute Profile) command support to access the data. However, one of the drawbacks is that, as far as we know, there is no commercial hardware with all the sensors that our design incorporates.

For this reason, it was decided to build the device to measure, taking into account the needs of our models.

The IMU is used as a pedometer and as a fall detector. The device described in this section has been divided into two parts: the first part is a harness which houses the ECG, EDA and IMU sensors. The second part is a bracelet in which a virtual assistant has been embedded, which is responsible for receiving the data of the harness.

This data is pre-processed by the bracelet which behaves as a bidirectional system, allowing the sending and receiving of data from the web-service. The data sent to the web service are analysed in depth using different techniques of artificial intelligence (AI) and the result obtained from this analysis is sent to the bracelet to inform the user.

**Figure 2:**
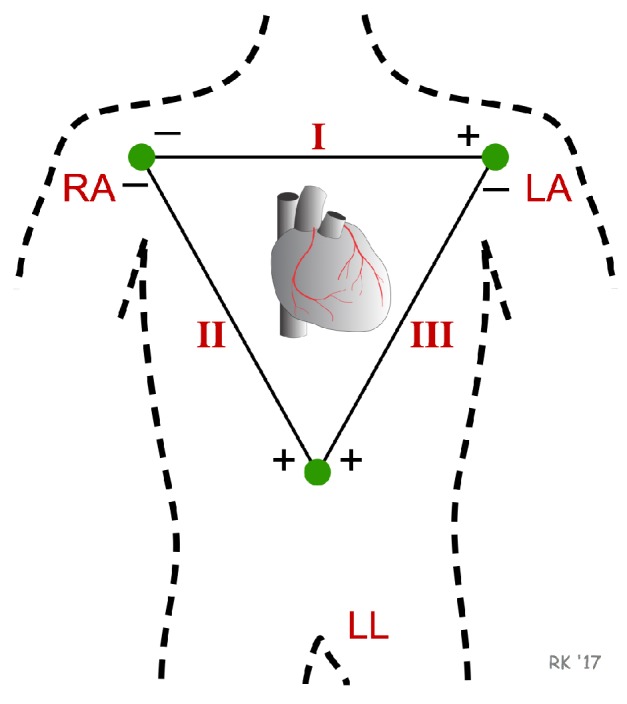
Einthoven triangle guide for positioning chest electrocardiogram leads (not to scale). RA—right arm; LA—left arm; LL—left leg [34].

To acquire these signals, the harness needs a communication interface between the skin and the capture device. This interface is achieved through electrodes, which are built with a gel that facilitates electrical conduction. In our case, we have decided to use stainless steel electrodes, which allows us to reuse the device and in turn facilitates its use. To acquire the heart signal, it is necessary that the electrodes form a triangle; this triangle is known as the triangle of Einthoven (Figure 2). This triangle allows us to capture the standard bipolar leads, which are the classic electrocardiogram leads. These leads record potential differences between electrodes located on different extremities.

**D1 or I:** Potential difference between right arm (RA) and left arm (RL). Its vector is in the 0° direction.
**D2 or II:** Potential difference between right arm (RA) and left leg (LL). Its vector is in the 60° direction.
**D3 or III:** Potential difference between left arm (RL) and left leg (LL). Its vector is in the 120° direction.

This electrical potential difference is in the range of millivolts (mv), a magnitude too small to be acquired by the data processing system. This is why it is necessary to amplify the signal from mv to volts (V). For this, the harness has an AD8232 instrumentation amplifier (Norwood, MA, USA) [35], which amplifies the potential difference up to 3.2 V, thus facilitating the signal acquisition process.

**Figure 3:**
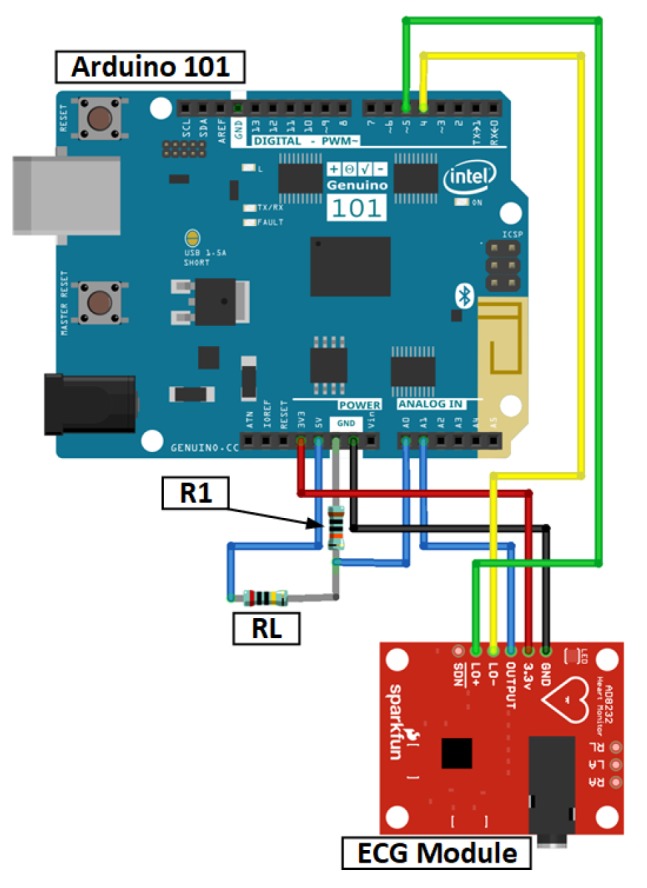
Main circuit of the chest strap data acquisition system, the cable connection points and resistors are highlighted for reproducibility.

In addition, the harness incorporates electrodes that allow for capturing the electrical activity of the skin (EDA); this signal can be acquired using different techniques. They can use operational amplifiers, instrumentation amplifiers, transconductance amplifiers, among others. In our case, we have decided to simplify the circuit to a voltage divider which is polarized with the battery voltage (3.3 V) (Figure 3); this voltage divider is composed of two resistors RL and R1. RL refers to the skin resistance which is in the order of Mega ohms and R1 (500 Kilo ohms) is a series resistance in which the voltage variation is measured.

**Figure 4:**
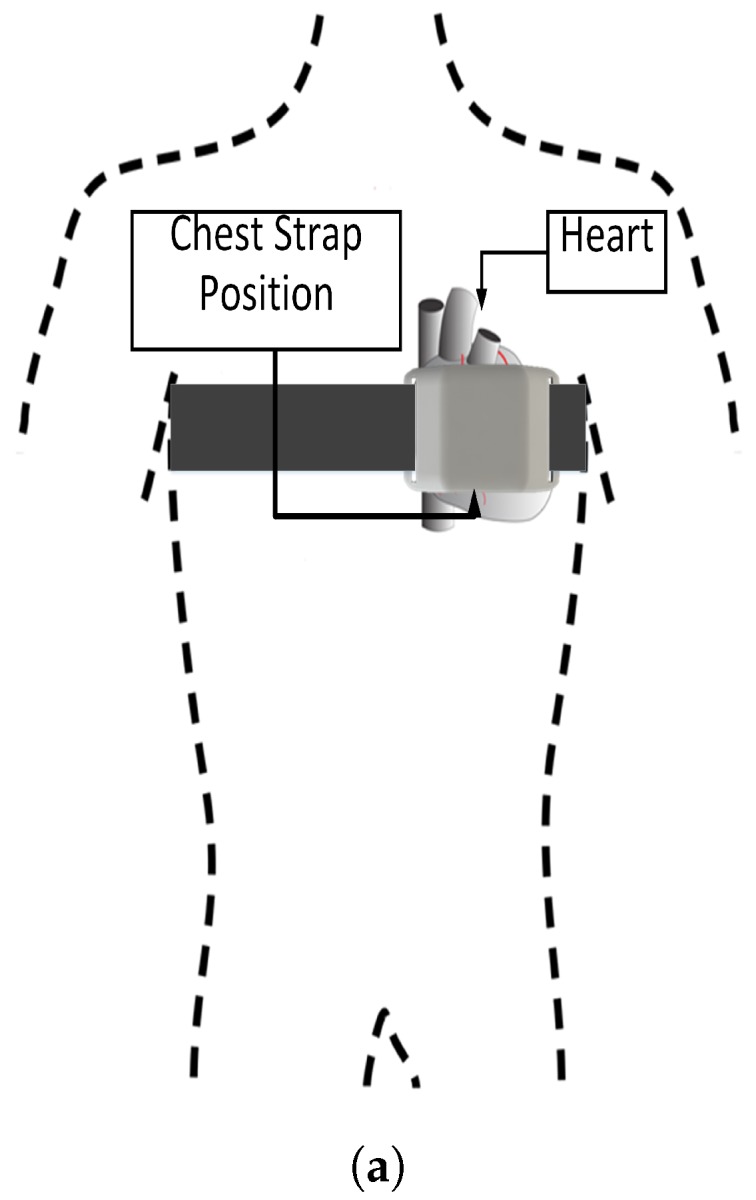

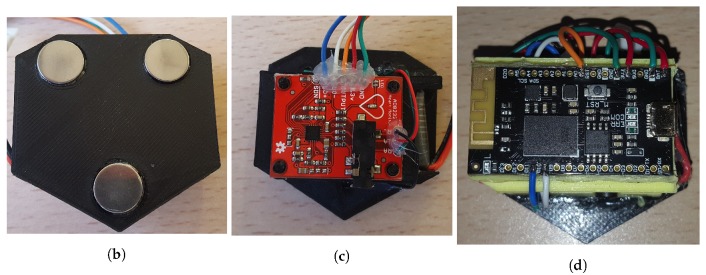
(**a**) Location of the chest strap. For better results, it has to be placed as centered over the heart as possible, the strap has to be tight but not constrictive (based on [34]). (**b**) View of the sensors’ leads (using the *Einthoven* triangle). (**c**) ECG (electrocardiography) module on the top side of the chest strap sensor system. (**d**) Arduino 101 that is placed above the ECG module and a small battery. Chest strap prototype (placement (**a**); 3D printed device (**b**–**d**)).

Once the two signals are in the required voltage ranges, it is necessary to convert these analog signals to digital. For them, the harness has a data acquisition system based on an Arduino-101 development system (see Figure 4 which shows different views of the chest strap prototype). This system has several converters from analog to digital or ADC, which allow for converting the continuous signal from the sensors to discrete value. The ADCs found within the Arduino-101 are 12 bits, which provides a conversion resolution of 0.0008 (Volts). In addition, the Arduino-101 incorporates a low-power Bluetooth communication system (BLE) and IMU, which is used as a fall detector and pedometer.

The data acquired and pre-processed by the Arduino-101 is sent to the wizard using BLE communication to perform this communication and the standard for the services of Generic Attributes (GATT). These are sets of characteristics and relationships with other services that encapsulate the behaviour of part of a device. A GATT profile describes a case of general use, functions, and behaviours based on GATT functionality, which allows for broad innovation while maintaining full interoperability with other Bluetooth® devices.

This standard describes a series of Universally Unique IDentifiers (UUIDs), which are used to identify services. These services can be viewed at [36] For our device, we have used two UUIDs offered by GATT, one for heart rate and one for RSC. The other services offered by the harness such as care detection and serial communication (UART) between devices; their UUIDs were generated using the following website. These UUIDs can be seen in Table 1 and Table 2.

Once the signals have been acquired and pre-processed, they are sent to the assistant located on the bracelet. This bracelet was built using the M5Stack-Fire [37] development system, which features a liquid crystal display (LCD), IMU, microphone, speakers and an ESP-32 chip [38]. This chip is widely used to make applications on the Internet of Things (IoT) and ambient intelligence (AIm). This is mainly due to its small size and to the fact that it incorporates communication protocols such as Wifi and Bluetooth. Once the wizard inside the bracelet receives the preprocessed information from the harnesses, it is sent to the web service for in-depth analysis. This analysis uses different AI tools to analyse the data and try to detect emotional states or stress.

**Figure 5:**
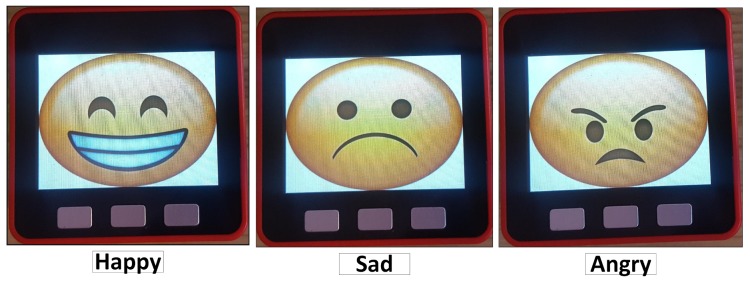
Visual representation example of EMERALD’s virtual assistant (designed for easy user understanding).

To display emotions, the virtual assistant (embedded inside the M5stack) is able to project images, called *faces*, that convey human-like emotions. The objective is to facilitate the interaction with the users. An example of the *faces* can be seen in Figure 5.

### 3.2. Software Description

The EMERALD inner workings is divided into two modules: the *Empathy Module* and the *FitCLA*. The *Empathy Module* goal is to perceive the current emotion of the user, while the *FitCLA* is an assistant that monitors, profiles and recommends exercises to the user.

#### 3.2.1. Empathy Module

The Cognitive Service is a new tool that uses a machine learning technique to create smarter and more engaging applications. This cognitive service introduces an API (Application Programming Interface) to detect emotion, speech recognition, conversion of text to speech and more. Some of the most important services that can be used right now are *Microsoft Cognitive Service (formerly Project Oxford)* (https://azure.microsoft.com/en-us/services/cognitive-services/), *IBM Watson* (https://www.ibm.com/watson/), *Google* (https://cloud.google.com/) and *Amazon AWS* (https://aws.amazon.com).

**Figure 6:**
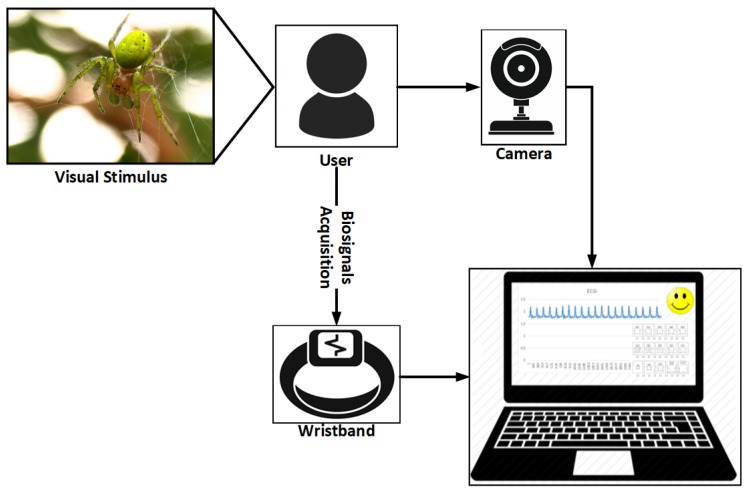
Description of the data capture process for the model training. The user is exposed to visual stimulus and their bio-signals along with facial expressions are recorded, classified and used to train the model.

One of the problems encountered is directly related to the generalization of the model, which allows us to detect emotion for any person. To try to make this generalization, an experiment was developed in which a group of individuals were subjected to different visual and auditory stimuli. During this process, four data sets were extracted. The first one comprises the personality, the emotion detected through a webcam, different bio-signals and the emotion that the user felt before each stimulus. The personality was extracted using the OCEAN test [39]. This test allowed us to group the subjects by their personalities. The second parameter set was formed by the emotions that the users expressed through the variations of their faces, when they were submitted to the stimuli. The third data set relates to the bio-signals (ECG, PPG, EDA) that were acquired during the stimulus. Finally, a subjective input, which was obtained through the SAM (Self-Assessment Manikin) test [40]. In this test, the user expressed the emotion he felt when the stimulus presented. This process can be seen in the following Figure 6. In order to obtain generalized models of emotion detection, a total of 150 people were used in a residence in the north of Portugal.

The cognitive service was divided into two parts: one part specialized in the recognition of emotions through image processing (capturing data through the camera) and the other part in which bio-signals are used to recognize emotions (capturing data through sensors). These elements are explained below.

To detect emotion through bio-signals, we used the DEAP (Database for Emotion Analysis Using Physiological Signals) [41] data set, which has a series of physiological signals associated with emotional states. The dataset was divided into three parts training, test and validation, for training 80% was used, for test 10% and validation 10%. The following Table 3 (Partial data can be downloaded from: https://upvedues-my.sharepoint.com/:u:/g/personal/jairina1_upv_edu_es/EceD-F-zfphGs_Uegbp2uc8B9P6bH1Zz4-IJvV1fWpoUcA?e=5xsa58) shows the distribution of data used in the experiments. This distribution is done randomly each time the training is performed.

**Figure 7:**
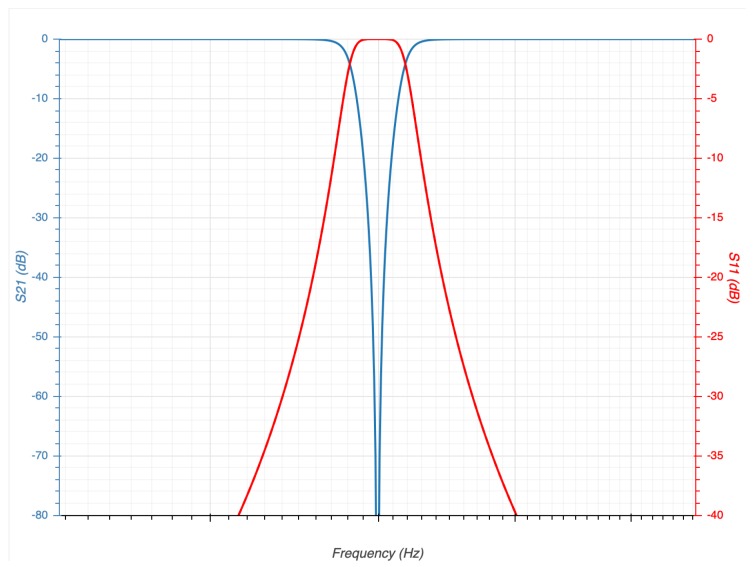
Bandstop filter response dB attenuation (with the features of Table 4).

This database incorporates signals such as electroencephalography, respiratory frequency, electrocardiography, electrical activity of the skin, among others. For our tests, we have decided to use only three of these signals (ECG, PPG and EDA), since they do not generate any kind of stress in the users unlike the EEG [42,43]. In this dataset, all the signals were filtered to eliminate the electrical noise of 50Hz; this is important to perform a good classification process. Our system performs pre-processing signals, for which a software *Butterworth* filter band-stop type was applied. The structure of our filtering design is detailed in Table 4 and the response of our filter can be seen in Figure 7.

This filter cuts the noise introduced by the electrical network; in addition, this filter architecture is the most used in applications of signal filtering [44].

Once a clean signal is obtained, the next step is to extract the statistical characteristic of each of the signals; six characteristics have been extracted from each of the signals as suggested by Picard et al. in [45]:

1. Mean
  (1) $$\mu =\frac{1}{N}\sum_{n=1}^{N}X_{n},$$
2. Standard Deviation
  (2) $$\rho =\sqrt{\frac{1}{N-1}\sum_{n=1}^{N}{\left(X_{n}-\mu \right)}^{2}},$$
3. Mean of the absolute values of the first difference (AFD)
  (3) $$AFD=\frac{1}{N-1}\sum_{n=1}^{N-1}\left|X_{n+1}-X_{n}\right|,$$
4. Mean of the normalized absolute values of the first difference (AFDN)
  (4) $$AFDN=\frac{1}{N-1}\sum_{n=1}^{N-1}\left|\tilde{X_{n+1}}-\tilde{X_{n}}\right|,$$
5. Mean of the absolute values of the second difference (ASD)
  (5) $$ASD=\frac{1}{N-2}\sum_{n=1}^{N-2}\left|\tilde{X_{n+2}}-\tilde{X_{n}}\right|,$$
6. Mean of the normalized absolute values of the first difference (ASDN)
  (6) $$ASDN=\frac{1}{N-2}\sum_{n=1}^{N-2}\left|\tilde{X_{n+2}}-\tilde{X_{n}}\right|.$$

The six characteristics, extracted by each signal (ECG, EDA, PPG), represent the inputs of the neural network. In this way, we have a neural network model with 18 inputs and seven outputs. These seven outputs correspond to the seven emotions that the system is able to recognize. The seven emotions to recognize are basic emotions, which are the same emotions to recognize using the camera. These seven emotions are the following: *Afraid*, *Angry*, *Disgusted*, *Happy*, *Neutral*, *Sad* and *Surprised*. The parameters of the best model obtained are represented in Table 5:

In the same way that the parameters of the network used to analyse the images were modified, the network that analyses the signal was modified to try to obtain the best results.

**Figure 8:**
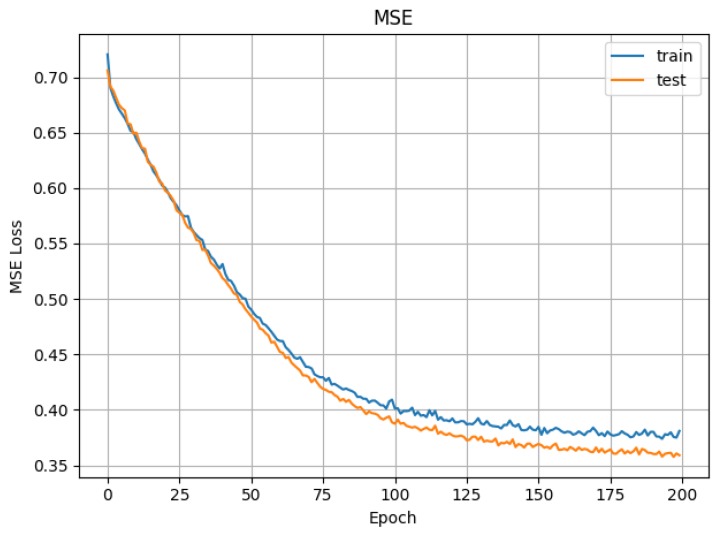
CNN (convolutional neural network) mean square error in training and test phases (training 80%, test 10%, validation 10%).

The mean square error obtained in the training and test phases are displayed in Figure 8. After the very first epochs, the values converge rapidly and remain constant almost linearly throughout the remaining epochs. The low values of the mean square error indicate that the training process is being done correctly (the attained values have low variation).

**Figure 9:**
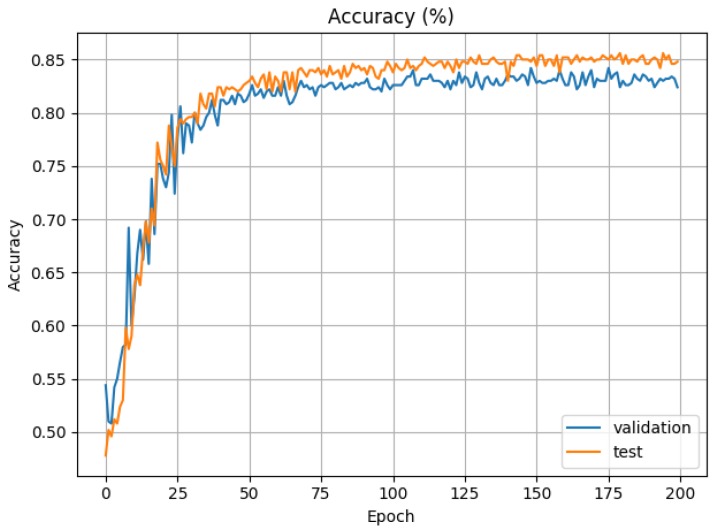
CNN accuracy in test and validation phases (training 80%, test 10%, validation 10%).

Figure 9 shows the accuracy (or precision) in the validation and test phases. We have achieved a stable 75% of accuracy in the validation process. This shows that, although it is possibly improvable, our current approach already produces relevant results.

**Figure 10:**
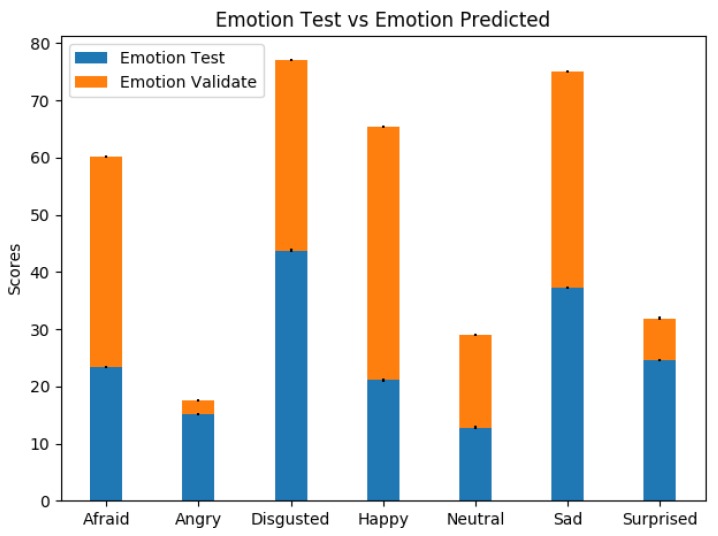
Comparison of test phase vs. validation.

Figure 10 shows a variability in the results obtained in the test phase vs. the validation phase. This variation is due to the fact that the users in the validation face were not static. If some type of movement was not performed, these movements can introduce erroneous data to the systems. When people move, electromyography signals (EMG muscle activity) are introduced into the ECG and, at the same time, these movements can affect the photoplethysmography signals by varying the measurement of PPM. This introduced noise is very difficult to eliminate and, to try to solve it, we have incorporated software filtering employed in [46,47].

We believe that, with optimized sensor systems (reducing the noise and data drop) and with a more robust CNN, we are able to achieve a higher accuracy and reduce the mean square error. We are currently working in improving the chest strap sensors to reduce the mean square error.

#### 3.2.2. FitCLA

The FitCLA is, in a broader sense, a cognitive assistant platform that aims to help people with cognitive and physical impairments (e.g., memory loss, assisted mobility) by reminding them about future and current events and connecting them with the caregivers for constant monitoring and suggesting playful exercises. It does this by establishing a monitoring environment (with the wristband) and using diverse visual interfaces (the visual assistant, webpages, etc.) to convey information to the users, while providing medical and general information to the caregivers.

The platform uses an interactive process of scheduling events and managing tasks that require little interaction from the users (caregivers and care-receivers alike), thus making the scheduling process simple. Furthermore, the FitCLA has an activities recommender that suggests to the care-receivers activities that have a physical and mental positive impact; this feature follows the active ageing effort. By engaging elderly people in activities (either alone or accompanied), their cognitive and physical functions are improved, and arguably most importantly, they are happier. For instance, there are several findings that simple group memory games helped contain the advance of Alzheimer’s [48,49].

The FitCLA is a development spun from the iGenda project [50,51,52], giving way to a robust platform that is interoperable with other systems. To this, the FitCLA is adjusted to currently only suggest exercises, which are the objective of this assistant, keeping the interaction simple with the users to observe their long-term adoption of the EMERALD.

The main components of the FitCLA are four: the agenda manager, the activities recommender, the module manager and the message transport system. Briefly explained, they have the following functionalities:

- The agenda manager keeps the information of each user (caregiver and care-receiver) updated and, upon receiving new events, it schedules them in the correct placement;
- The activities recommender regularly fetches an activity that the user enjoys performing and is appropriate according to the health condition (often people enjoy activities that are not physically or mentally advised) and schedules them in the user’s free time. It is evolutionary, as it adapts to the user’s likes (by the user acceptance or refusal of the suggestion) and to the current medical condition, e.g., if the user has a broken leg the platform refrains from suggesting walking activities;
- The module manager is the gateway for coupling new features and communicating with the different agents.
- The message transport system establishes the API of the system. It is the door to the platform internal workings.

Being more specific in terms of the activities recommender, it uses an internal algorithm that optimizes the activities selection procedure. Each user has a profile in the system, consisting of a table of the personal and medical information, with fields like: “is able to perform hard physical activities”, “should/arms conditioning”. These fields are used to access the user ability to perform certain activities. Each activity is categorized in groups that detail their physical/cognitive impact. For instance, the activity *Light gardening* is ranked with cognitive impact, with mild impact to shoulders, arms, column and light impact to legs and feet, and using dangerous tools. Meaning that a person with shoulders, arms and column problems would not be advised (minding the level of impairment each user has), neither people with hand problems nor high cognitive disabilities (due to the dangerous tools usage). Each problem is factored in a straightforward algorithm (showed in Algorithm 1) that filters the activities.

**Algorithm 1:** FitCLA selection algorithm

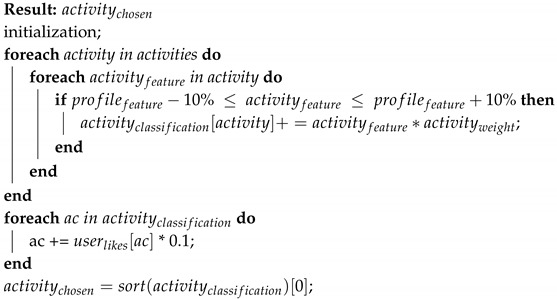



For each activity feature, a weight of importance (at the moment, all weights are the same) is multiplied and summed to the activity classification. The maximum value that can be obtained is 0.9, leaving the remainder 0.1 to the user preference. These values are configurable to each user (meaning that one user may have a higher percentage on their preference), these values being the initials. All of the weights can be changed by the caregivers.

Thereon, the users are inquired at the end of the suggestion if they agree with the suggestion. The response is factored in the weight of the user likes to each activity. This means that the system is steadily learning the preferences of each user. Thus, being slowly optimized to respond to each user preference and needs. Finally, the FitCLA has thresholds that, when achieved, result in either notifying the caregiver of slightly nudging the weights. For instance, in a normal setting, if a user denies activities that closely fit the health profile seven times, the maximum weight of the activity features is decreased and the user likes is increased, the system being able to re-weight the values if this "nudge" is not enough. The caregiver is notified if abnormal behaviours (like the negation of several activities) are reached.

Currently, the FitCLA assumes that, upon acceptance of the users to perform exercises, they truly perform them, thus being the data captured during that period cross-linked with those exercises.

The FitCLA is, at its core, a multi-agent system. Thus, new features are implemented in the form of new agents. Thus, the module manager is an archive of features of each agent/module and, when any agent/module requires a feature, the module manager responds with the agent/module identification, as well as their address and API structure. This component was built to streamline the connection of foreign systems to the platform. It was designed to not only communicate with other digital agents, but also with visual clients (smartphones, robots, televisions).

The FitCLA tightly integrates with the hardware available (wristband, chest strap, and virtual assistant) and provides information about each user schedule and forwards recommended activities to the virtual assistant. Moreover, it receives the information from the hardware platform and uses that information to change the recommendation parameters (as a post-process response to the recommendation) influencing positively or negatively in accordance to the emotional response. The way it does this is using the internal classification of the exercises: high-intensity exercises are classified as emotional boosters, and low-intensity exercises are classified as emotional de-stresser.

Apart from this, the FitCLA is able to provide information to the caregiver about the interactions of the users and their emotional status, allowing them to make informed decisions about the ongoing treatments.

We present a functional example below of the EMERALD operation of both software and hardware components.

### 3.3. EMERALD Example

In this section, we present an example that shows the operation of EMERALD and how the user is able to interact with it. The goal is to demonstrate the discreteness of the devices and the transparent operation of the software, prompting the user’s attention only when required.

**Figure 11:**
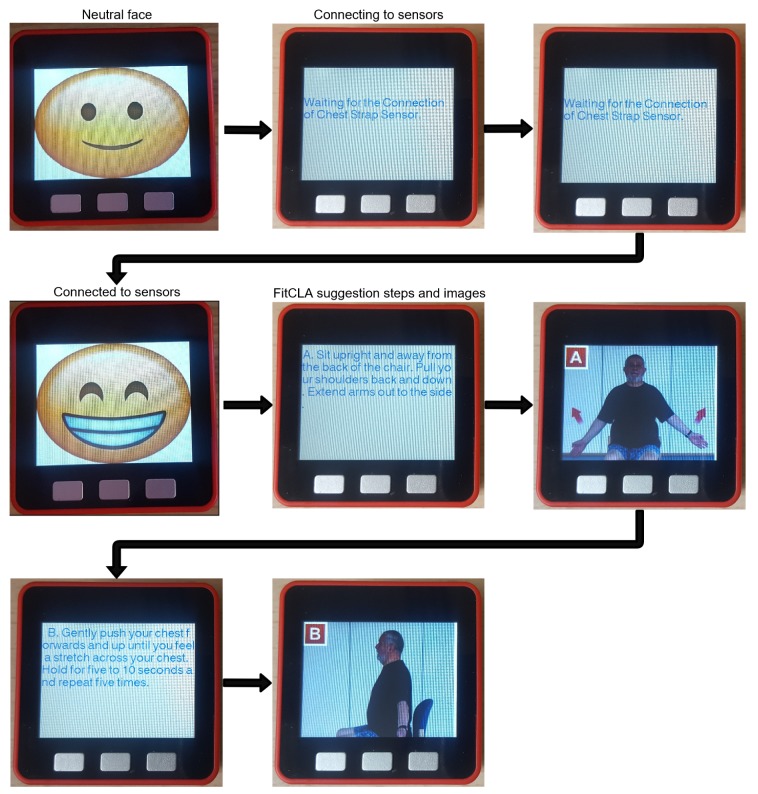
Virtual assistant emotion display and a FitCLA suggestion. The VA is waiting for the sensor system synchronization; when scheduled, the screen is used to give information (description and exemplifying images) of the exercise to be performed (in this case the *Arms raise* example [53]).

The assistant (in this case its visual form), present in the bracelet, was designed to be appealing and fun to interact. To achieve this, a cartoonish face was used. It shows some emotional states and human-like expressions to convey meaningful visual engagement to accompany the messages presented. The bracelet also possesses a loudspeaker that can be used to transmit audio information and it visually displays the suggestions of the FitCLA in the screen (see Figure 11).

The EMERALD operation flux is described in the following steps:

1. The user is registered on the EMERALD system and his/her profile is introduced in the platform as a starting point (defining the user physical/cognitive limitations). After this, the user is fitted with the bracelet and the harness and the system is activated.
2. EMERALD is on *standby* mode, meaning that the hardware and low-level operation is active. Thus, the *Empathy Module* is operational and constantly measures the emotional state of the user.
3. The bracelet shows *faces* that convey the EMERALD’s emotion, which is based on the information of sensors and the historic evolution of the system. For instance, if the ECG and EDA/IMU levels are high (meaning that the user is excited), the bracelet shows a surprised/concerned *face* to show the user that something is wrong, creating an emotional bond that should engage the user into keep calm. The aim is to create an emotional connection, and, like humans, if someone cares for one’s emotional and physical state, it is common that their emotional stance is more calm and collected as to not create concern.
4. The information about the emotional state is made available to the FitCLA, which in turn optimizes the suggestion of activities to better suit the emotional state. For instance, if the user is excited, the FitCLA may suggest a ten minute session of yoga or gardening (according to each user abilities). The FitCLA uses a boost/counteract algorithm that promotes positive emotional states and counteracts negative emotional states.
5. The user receives the exercises/activities notification and information in the bracelet (see Figure 11) at specific or periodic scheduling times (pre-configured and changeable).

Figure 11 exemplifies the EMERALD operation flux. The assistant, when in standby, displays its face. Then, when the FitCLA prompts an exercise to the user, it first displays a text instruction of the exercise, accompanied by a voice explanation; then, it displays images explaining the correct body position used on the exercise (rightmost part of Figure 11). These images are presented to users that have a medium-to-high level of cognitive abilities and some motor skills. The exercises used were designed to be performed by elderly people without assistance of caregivers by the British National Health Security (NHS) [53], for which they are absolutely confident by being validated by medical experts. Nonetheless, we try to adjust the exercises to each user according to their physical limitations. Currently, due to hardware limitations, the bracelet is only able to play pre-recorded messages, but it has a sufficiently large memory to withstand all necessary voice instructions.

We are aware that the size of the screen limits the quantity and quality of the information presented and we tried to overcome this issue with the audio instructions. Nonetheless, in future iterations, we will aim to interact with home devices (such as Smart TVs) to display pictures and/or videos of high definition instructions of the exercise.

## 4. Conclusions and Future Work

A cognitive assistant platform that aims to help people for active ageing has been presented in this paper. EMERALD generates and adapts personalized exercises sequence for an individual—in this case, an elderly person at his/her home. To do this, the system incorporates a set of bio-sensors integrated in a chest strap and in a wristband. These sensors capture information that can be measured in the form of physical stress while a user is doing exercises; it is also able to perceive the emotion of the person.

This information is employed by FitCLA to adapt the recommended exercises to each user. This allows for an adequate exercises sequence (as the FitCLA is able to propose one or a batch of exercises) according to the physical state of the person and to the evolution of his/her emotions. Moreover, EMERALD is able to provide this information to the caregivers, allowing them to take action, changing their activities or treatments or proposing new ones, or just monitor their physical/emotional progression.

In terms of results, EMERALD is being currently tested in a daycare centre in the northern area of Portugal named *Centro Social Irmandade de S. Torcato* and other tests will be implemented at different centers for people with disabilities, under the RISEWISE project. At the moment, tests are being performed on a small number of patients at the *Centro Social Irmandade de S. Torcato* under the supervision of the caregivers. In order to obtain some kind of initial feedback, a simple questionnaire has been made by workers (caregivers: registered nurses and medical personnel) of the daycare centre. The questionnaire was responded to by only 10 persons, which is the number of people working in the centre. In Table 6, a summary of the questions performed is presented. In terms of the utility and adequacy of the suggested activities for the residents, the responses seem to be very positive. Nevertheless, these are very preliminary data. Until now, the tests are limited to the collection and analysis of the data generated by recommending simple exercises. Although they are still too incipient to be presented in this paper, these tests show positive results, pointing out the fact that the development of the platform is proceeding in the right direction.

In terms of people with disabilities, we will begin testing EMERALD and observe if it is well adopted by them, and adjust accordingly if not. We believe that the way that EMERALD interfaces with the users is simple and intuitive; thus, we think that the usage by people with cognitive disabilities will not feel difficulties.

As future work, apart from the development of more intensive tests, it is proposed to integrate other types of sensory devices such as cameras in order to analyse whether the proposed exercises are being carried out satisfactorily and to compare the results with the patient’s emotional state and stress.

## Figures and Tables

**Table 1:** Table of Bluetooth UUID (Universally Unique IDentifier) standard identifiers for reproducibility.

Name	Uniform Type Identifier	Assigned Number
Heart Rate	org.bluetooth.characteristic.heart_rate_measurement	0x2A37
RSC Measurement	org.bluetooth.characteristic.rsc_measurement	0x2A53

**Table 2:** Table of Bluetooth UUID communication identifiers for reproducibility.

Name	Assigned Number
UART Rx	0xDFB2
UART Tx	0xDFB1
Fall Detection	0xDFB3

**Table 3:** Data distribution.

Total Raw Data	Features Extracted	Training Partition	Test Partition	Validation Partition
866 (per signal)	6 (per signal)	710	78	10

**Table 4:** Filter and features used for pre-processing the bio-signals.

Response	Filter Type	Order Filter	Lower Cutoff Frequency	Upper Cutoff Frequency
Bandstop	Butterworth	3	48	52

**Table 5:** Configuration parameters of the selected network.

**L2 Regularization or l2-penalty**	0.01
**Hidden Layers**	[32, 64, 128, 64, 32]
**Dropout Rate**	0.2
**Monitor**	val_loss
**Min Delta**	10

**Table 6:** System evaluation.

	Strongly Agree	Agree	Undecided	Disagree	Strongly Disagree
Would the proposed system be useful and would it improve your work?	10%	50%	30%	10%	0%
Do you think the system has improved the activity of the residents?	10%	60%	30%	0%	0%
Would this system give useful information about the residents and activities that are doing?	20%	40%	20%	20%	0%
